# Establishment of a novel NFAT-GFP reporter platform useful for the functional avidity maturation of HLA class II-restricted TCRs

**DOI:** 10.1007/s00262-023-03420-8

**Published:** 2023-03-20

**Authors:** Fumihiro Fujiki, Soyoko Morimoto, Yuya Nishida, Satoe Tanii, Nao Aoyama, Miki Inatome, Kento Inoue, Akiko Katsuhara, Hiroko Nakajima, Jun Nakata, Sumiyuki Nishida, Akihiro Tsuboi, Yoshihiro Oka, Yusuke Oji, Shinji Sogo, Haruo Sugiyama

**Affiliations:** 1 Department of Cancer Immunology, Osaka University Graduate School of Medicine, Suita, Osaka 565-0871 Japan; 2 Department of Cancer Immunotherapy, Osaka University Graduate School of Medicine, Suita, Osaka 565-0871 Japan; 3 Department of Cancer Stem Cell Biology, Osaka University Graduate School of Medicine, Suita, Japan; 4 Department of Clinical Laboratory and Biomedical Sciences, Osaka University Graduate School of Medicine, Suita, Japan; 5 Department of Functional Diagnostic Science, Osaka University Graduate School of Medicine, Suita, Japan; 6 Strategic Global Partnership & X (Cross)-Innovation Initiative, Graduate School of Medicine, Osaka University & Osaka University Hospital, Suita, Japan; 7 Department of Respiratory Medicine and Clinical Immunology, Osaka University Graduate School of Medicine, Suita, Japan; 8 Department of Research Management, Otsuka Pharmaceutical Co., Ltd, Tokushima, Japan; 9 Joint Research Chair of Immune Therapeutic Drug Discovery IFReC, Osaka University Graduate School of Medicine, Suita, Japan

**Keywords:** CD4^+^ T cell, TCR, HLA class II, WT1, TCR-T therapy, NFAT-GFP reporter

## Abstract

CD4^+^ T cells that recognize antigenic peptides presented on HLA class II are essential for inducing an optimal anti-tumor immune response, and adoptive transfer of tumor antigen-specific TCR-transduced CD4^+^ T cells with high responsiveness against tumor is a promising strategy for cancer treatment. Whereas a precise evaluation method of functional avidity, an indicator of T cell responsiveness against tumors, has been established for HLA class I-restricted TCRs, it remains unestablished for HLA class II-restricted TCRs. In this study, we generated a novel platform cell line, CD4-2D3, in which GFP reporter was expressed by NFAT activation via TCR signaling, for correctly evaluating functional avidity of HLA class II-restricted TCRs. Furthermore, using this platform cell line, we succeeded in maturating functional avidity of an HLA class II-restricted TCR specific for a WT1-derived helper peptide by substituting amino acids in complementarity determining region 3 (CDR3) of the TCR. Importantly, we demonstrated that transduction of an avidity-maturated TCR conferred strong cytotoxicity against WT1-expressing leukemia cells on CD4^+^ T cells, compared to that of its original TCR. Thus, CD4-2D3 cell line should be useful not only to evaluate TCR functional avidity in HLA class II-restricted TCRs but also to screen appropriate TCRs for clinical applications such as cancer immunotherapy.

**Supplementary Information:**

The online version contains supplementary material available at 10.1007/s00262-023-03420-8.

## Introduction

CD4^+^ helper T cells (Th cells) are essential effector cells for eliciting a potent anti-tumor immune response. Th cells provide so- called “help”, such as a variety of cytokines and cell-to-cell signals to other immune cells including CD8^+^ cytotoxic T lymphocytes (CTLs) and dendritic cells (DCs) [1, 2]. In the absence of CD4 help, it is difficult to induce tumor-associated antigen (TAA) -specific CTLs due to a failure of cross-presentation in “helpless” DCs. Furthermore, helpless CTLs are easy to occur activation-induced cell death (AICD) on re-encounter with antigen [3]. In addition to the helper functions, CD4^+^ T cells also exert cytotoxicity against tumor cells via Fas/FasL and perforin/granzyme pathway [4–7]. Accordingly, increasing TAA-specific CD4^+^ T cells, for example, by vaccination or adoptive cell-transfer has been thought to be crucial to eradicate cancer. In particular, the adoptive transfer of TCR gene-engineered T cells (TCR-T) would be a promising strategy for increasing TAA-specific CD4^+^ T cells in cancer patients [8].

In clinical and preclinical studies of adoptive TCR-T therapy, TCR avidity and TCR functional avidity have been a determinant of in vitro/in vivo anti-tumor response in TCR-T cells [9–11]. Therefore, selecting optimal-avidity TCRs from numerous candidate TCRs is key for success in adoptive TCR-T therapy. In general, TCR avidity is measured with peptide/MHC (pMHC) tetramers, and TCR functional avidity is assessed using a titrated concentration of antigen peptide with antigen-presenting cells. Unfortunately, available pMHC tetramers for HLA class II-restricted TCRs (hereafter, HLA class II tetramers) are largely limited, compared to those for HLA class I-restricted TCRs, due to a diversity of HLA class II. In addition, some studies have reported no correlation between reactivity (binding intensity) to tetramers and T cell function [12, 13]. Accordingly, TCR functional avidity is broadly used as an indicator for selecting the most promising HLA class II-restricted TCRs. Whereas TCR affinity is an absolute index, TCR functional avidity is a relative index because it is easily influenced by various factors such as the expression level of co-receptors (CD8/CD4) and TCR clustering. For accurate assessment of TCR functional avidity, therefore, primary T cells are an inappropriate platform because they are heterogeneous and express endogenous TCRs that cause incorrect TCR clustering by mispairing with transduced TCRs [14] and competing for CD3 molecules [15]. However, in many studies, primary T cell has been used for the assessment, which makes difficult the comparison of TCR functional avidity across studies. Thus, a standardized platform cell was demanded.

One of the most important criteria for defining a platform cell is whether or not the platform cell can predict responsiveness of TCR-transduced T cells. Accordingly, TCR functional avidity evaluated by using a bona-fide platform cell correlate with actual responsiveness and function of the TCR-transduced T cells. Only under this relationship, we can be exempted from experiments using primary human T cells to screen for optimal or high-avidity TCRs. Although many researchers have established Jurkat cell-based cell lines useful for checking function of isolated TCRs, the relationship in these cell lines have not been addressed. For HLA class I-restricted TCRs, we previously established an endogenous TCR-null platform cell line, named 2D3 that has NFAT-GFP reporter system [16]. In 2D3 cells, TCR functional avidity was precisely and efficiently evaluated by the expression of GFP reporter gene driven by NFAT activation via TCR signaling. Importantly, the functional avidity correlated with CD8^+^ T cell function such as cytokine production, proliferation, and cytotoxicity. Consequently, this cell line is expected to be a standardized platform cell for selecting optimal HLA class I-restricted TCRs for TCR-T therapy. Recent studies have demonstrated that HLA class II-restricted TCRs with high reactivity (avidity) to TAAs are also promising tools for the therapy because TAAs-reactive CD4^+^ T cells play essential roles in tumor eradication [8, 17, 18]. Nevertheless, no platform cell line for HLA class II-restricted TCRs was established so far. In this study, we have generated CD4-2D3 cell line by transducing 2D3 cell line with human CD4 gene and have demonstrated that this cell line should be a useful platform cell line for evaluating functional avidity of HLA class II-restricted TCRs. Furthermore, using this cell line, we accomplished functional avidity maturation of an HLA class II-restricted TCR specific for a WT1-derived helper peptide by substituting amino acids in CDR3 of the TCR. Importantly, the transduction of an avidity-maturated TCR-induced stronger cytotoxicity in CD4^+^ T cells. Thus, CD4-2D3 cell line should be useful not only to evaluate TCR functional avidity in HLA class II-restricted TCRs but also to screen appropriate TCRs for clinical applications such as cancer immunotherapy.

## Materials and methods

### Cell lines and human CD4^+^ T cells

TCR-null and NFAT-GFP reporter-expressing cell line, 2D3 [16], and HLA-DRB1*04:05/HLA-DPB1*0501-positive Epstein-Barr virus–transformed B cell line, B-LCL [19] were generated previously. C2F8-CIITA cell line was generated by the transduction of CIITA (XM_011522484) encoding pIRESpuro3 vector (Takara Bio, Otsu, Japan). These cell lines including K562 cell line [16] were cultured in RPMI 1640 (Nacalai Tesque, Kyoto, Japan) with 10% heat-inactivated FBS (SIGMA, St Louis, MO) and 1% penicillin/streptomycin (Nacalai Tesque). Lenti-X™ 293 T cell line was purchased from Clontech/Takara Bio (Otsu, Japan) and was cultured in DMEM containing 4.5 mg/ml glucose (Nacalai Tesque) with 10% heat-inactivated FBS. Human CD4^+^ T cells were isolated from peripheral blood mononuclear cells (PBMCs) using the magnetic BD IMag Cell Separation System according to the manufacturer’s instructions (BD Biosciences) after written informed consent was obtained from HLA-DPB1*05:01-positive healthy volunteers. T cells were cultured in X-VITO™ 15 (Lonza, Walkersville, MD) supplemented with 10% human AB serum (Gemini, West Sacramento, CA) and 40 IU/ml IL-2 (Imunace 35, Shionogi & Co., LTD).

### Antibodies, WT1 peptides, and reagents

A WT1-derived HLA class II-restricted helper peptide, WT1_332_ (KRYFKLSHLQMHSRKH) was purchased from PEPTIDE INSTITUTE INC. (Osaka, Japan). For flow cytometric analysis, the following mAbs were used: anti-TCR *α*/*β*-PE (IP26), anti-IFN-γ-PE (4S.B3), and anti-TNF-*α*-APC (MAb11) purchased from eBioscience (San Diego, CA) and anti-CD4-APC-H7 (SK3), anti-Perforin-APC (dG9), anti-Granzyme B-PE (GB11), and mouse IgG1κ isotype control-PE (MOPC-21) purchased from BD Biosciences and anti-CD3-PE-Cy7 (SK7) and mouse IgG2bκ isotype control-APC (MPC-11) purchased from BioLegend (San Diego, CA) and anti-CD4-APC-Cy7 (RPA-T4) purchased from Tonbo Biosciences (San Diego, CA). Anti-CD3 mAb (OKT-3, Tonbo Biosciences) and anti-CD28 mAb (CD28.2, Tonbo Biosciences) were used at concentrations of 2 μg/ml to stimulate human CD4^+^ T cells.

### Establishment of CD4-2D3 cell line

Human CD4 gene (NM_000616.4) was amplified using the following primers: CD4-F; 5’-TCG GCA AGG CCA CAA TGA ACC GGG GAG T-3’, CD4-R; 5’-CTG GCC TCG TGC CTC AAA TGG GGC TAC ATG-3’. The amplified product was cloned into pTA2 vector (TOYOBO, Osaka, Japan) and subcloned into XhoI-BamHI sites of pcDNA3.1(+) vector (Invitrogen, Carlsbad, CA). 2D3 cells were electroporated with the CD4 gene-encoding pcDNA3.1(+) and then 2D3 cells highly expressing CD4 were sorted several times. We obtained a highly and stably CD4-expressing 2D3 cell line in which GFP protein was expressed after TCR-stimulation and established it as CD4-2D3 cell line.

### Lentivirus preparation and generation of TCR-transduced cells

Preparation and transfection of Lentivirus were performed as described previously with minor modifications [4]. In brief, Lenti-X™ 293 T cells were co-transfected with CSII-EF-MCS-IRES2-Venus encoding TCR gene or empty plasmid, pCAG-HIVgp, and pCMV-VSVG-RSV-Rev (kindly provided by Dr Hiroyuki Miyoshi and Dr Atsushi Miyawaki, RIKEN). The supernatant containing lentiviruses was collected and concentrated and the concentrated viruses were stored at −80 °C until use. For lentiviral transduction, cells (activated CD4^+^ T cells, 2D3 cells, and CD4-2D3 cells) were spin-infected in the presence of recombinant lentiviruses and 10 μg/ml polybrene (Sigma) on RetroNectin (Takara Bio) -coated 48-well plate. The transduced CD4^+^ T cells (Venus^+^ cells) were sorted, expanded for 1 week by the stimulation with anti-CD3/CD28 mAbs or irradiated WT1_332_-pulsed autologous PBMCs, and then used for experiments. The transduced-2D3 and -CD4-2D3 cells were examined for TCR, CD3, and Venus expression by flow cytometry and then purified into Venus^+^ cells for NFAT-GFP reporter assay.

### Alanine-scanning mutagenesis of Clone 10-TCR

Site‐directed mutants of Clone 10-TCR for alanine‐scanning were prepared using KOD ‐Plus‐ Mutagenesis Kit (TOYOBO) with site‐specific primers. The site-specific primers are listed in Table 1. Clone 10-TCR (*α*-P2A-*β* cassette) -encoding pTA2 plasmid was used as a template. To generate S117A/H122A-TCR construct, S117A-TCR-encoding pTA2 plasmid was used as a template for inverse PCR by KOD ‐Plus‐ Mutagenesis Kit. The site-specific primers for this TCR were as follows: Forward primer; 5’- GCC TTT GGT GAT GGG ACT CGA CTC TCC ATC -3’, Reverse primer; 5’- CTG GGG CTG ATC GGC CGC CC -3’. Mutant TCR cassettes were subcloned into CSII-EF-MCS-IRES2-Venus vector for generating lentiviruses.

**Table 1:** Perimers for alanine-scanning mutagenesis

Mutant name	Position	Sequence (5′ to 3′)
S112A	Fwd	gccacggcaggggcgagcgatcag
	Rev	ggcacagaagtacacagatgtctctgggagg
T113A	Fwd	gccgcaggggcgagcgatcagcc
	Rev	gctggcacagaagtacacagatgtctgg
G115A	Fwd	gccgcgagcgatagccccagcatttt
	Rev	tgccgtctggcaccagaagtacac
S117A	Fwd	gccgatcagccccagcattttggtgtatggg
	Rev	cgcccctgccgtctggcac
D118A	Fwd	gcccagccccagcattttggtgatggga
	Rev	gcccccccagcattttggtgatgggactcg
Q119A	Fwd	atcgctcgcccctgccgtgct
	Rev	gcccagacattttggtgatgggactcgactc
P120A	Fwd	ctgatcgctcgcccctgccgt
	Rev	gcccattttggtgatgggactcgactctcc
Q121A	Fwd	gggctgatcgctcgcccctg
	Rev	gggctgatcgctcgcccctg
H122A	Fwd	gcctttggtgatgggatcgactctccatc
	Rev	ctggggctgactcgccc

### Clone 10-TCR mutation library

To generate Clone 10-TCR mutation library, Clone 10-TCR-encoding pTA2 plasmid was used as a template for inverse PCR by KOD ‐Plus‐ Mutagenesis Kit. The degenerate primers were as follows: Forward primer; 5’- GCC CCA GNN STT TGG TGA TGG GAC TCG ACT CTC CAT -3’, Reverse primer; 5’- TGA TCS NNC GCC CCT GCC GTG CTG GCA CAG AA -3’. The inverse PCR product were self-ligated, treated with DpnI enzyme, and then transformed into a high-efficient competent cells, HST02 (Takara Bio). After confirming the library size and amplifying in a liquid culture, plasmids were extracted, and then mutant TCR cassettes were cut out from the plasmids by BamHI (TOYOBO) and NotI (TOYOBO) and were inserted into CSII-EF-MCS-IRES2-Venus vector for generating lentiviruses.

### NFAT-GFP reporter assay

NFAT-GFP reporter assay was performed as described previously [16]. In brief, 1 × 10^5^ B-LCL cells and 1 × 10^5^ TCR-transduced CD4-2D3 cells were co-cultured at a 96-well plate in the presence of titrated concentrations of WT1_332_ for 16 h. The cells were washed with PBS (Nacalai Tesque) with 2% FBS and then measured for the frequency of GFP-positive cells in Venus-positive cells using a FACSAria instrument (BD Biosciences). Data were analyzed using FlowJo software (FlowJo, LLC).

### Flow cytometry analysis and intracellular cytokine detection assay

Cells were washed twice with PBS with 2% FBS (FACS buffer) and stained with various fluorescent dye-conjugated mAbs at 4 °C for 20 min. After the incubation, the cells were again washed twice with FACS buffer and then analyzed with FACSAria after the addition of 7-AAD (BioLegend) or SYTOX Blue (Thermo Fisher Scientific, Eugene, OR) for detecting dead cells. For intracellular cytokine staining, TCR-transduced CD4^+^ T cells (1 × 10^5^ cells) were co-cultured with autologous PBMCs (1 × 10^5^ cells) in the presence of 10 μg/ml of Brefeldin A (Sigma) and 20 μg/ml or titrated concentrations of WT1_332_ for 5 h. After cell surface marker staining, intracellular cytokine staining was performed as described previously [4].

### Detection of Perforin and Granzyme B

After TCR-transduction, TCR-transduced (Venus^+^) CD4^+^ T cells (1 × 10^6^ cells) were weekly stimulated with WT1_332_ (20 μg/ml) -pulsed, 30 Gy-irradiated autologous PBMCs (2 × 10^6^ cells). Three days later from the second stimulation, the cells were harvested, washed twice, and then used for intracellular cytokine staining without any re-stimulation.

### CD107a degranulation assay

TCR-transduced CD4^+^ T cells (1 × 10^5^ cells) were incubated with WT1_332_ (20 μg/ml) -pulsed or -unpulsed B-LCL (1 × 10^5^ cells) in the presence of 2 μM BD GolgiStop (BD Biosciences) and anti-CD107a-APC (H4A3, BD Biosciences) mAb for 5 h. Then, the cells were harvested, stained with anti-CD4-APC-Cy7, and analyzed by flow cytometry.

### ^51^Cr release assay

^51^Cr release assays were performed as previously described [20].

### Statistics

Data were analyzed using Prism 8 and 9. Normally and non-normally distributed data were analyzed by parametric (unpaired *t*-test) and non-parametric (Mann–Whitney test and Wilcoxon test) tests, respectively.

## Results

### Establishment of CD4-2D3 cell line for the evaluation of HLA class II-restricted TCRs

**Fig. 1:**
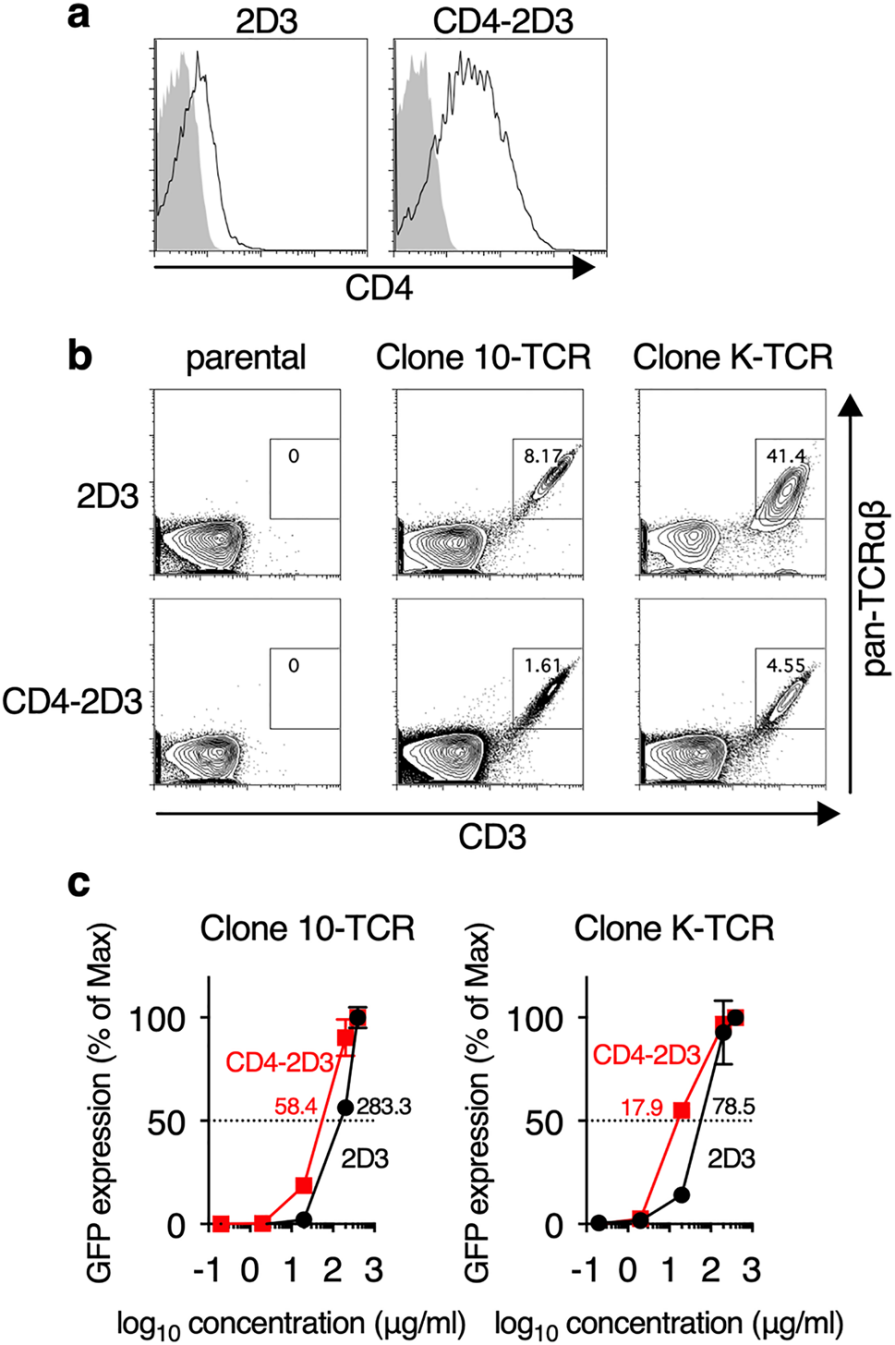
CD4-2D3 cells highly expressed GFP after TCR-stimulation. **a** Histograms showing CD4 expression levels in 2D3 and CD4-2D3 cells. **b** Expression of surface CD3 and TCR in HLA class II-restricted WT1_332_-specific TCRs (Clone 10-TCR or Clone K-TCR) -transduced 2D3 and CD4-2D3 cells. Non-transduced 2D3 and CD4-2D3 cells were used as a control (parental). Representative dot plots are shown. **c** HLA class II-restricted WT1_332_-specific TCRs (Clone 10-TCR or Clone K-TCR) -transduced 2D3 and CD4-2D3 cells were stimulated with the indicated concentrations of WT1_332_ helper peptide for 16 h. Representative peptide dose–response curves and their EC50 (numerical values) are shown

We previously established a 2D3 cell line whose GFP expression is induced by NFAT activation via TCR signaling and showed that this cell line was a useful tool for the evaluation of functional avidity of HLA class I-restricted TCRs [16]. However, the 2D3 cell line was not suitable for the evaluation of functional avidity of HLA class II-restricted TCRs because it did not express enough CD4 molecules to assist the interaction between the TCRs and HLA class II/antigen complexes (Fig. 1a *Left*). Therefore, we transduced 2D3 cells with human CD4 gene by electroporation and established a CD4-2D3 cell line, which stably and highly expresses CD4 molecule (Fig. 1a *Right*). To confirm that the CD4-2D3 cell line was useful to evaluate the functional avidity of HLA class II-restricted TCRs in comparison with 2D3 cell line, we separately transduced Clone 10- and Clone K-TCR that recognize WT1_332_ helper peptide in an HLA-DPB1*05:01- and HLA-DRB1*04:05-restricted manner, respectively [4, 21], into 2D3 and CD4-2D3 cell lines and established Clone 10-TCR- or Clone K-TCR-transduced 2D3 and CD4-2D3 cells. These Clone 10-TCR-transduced 2D3 and CD4-2D3 cells and Clone K-TCR-transduced ones showed co-expression of both TCR and CD3 molecule on their cell surface, indicating the correct expression of these transduced-TCRs (Fig. 1b). Next, we investigated whether CD4-2D3 cells were superior to 2D3 cells for detecting TCR signaling through transduced TCRs. As expected, the Clone 10-TCR-transduced CD4-2D3 cells expressed GFP more sensitive than the Clone 10-TCR-transduced 2D3 cells in response to WT1_332_ peptide stimulation (Fig. 1c, *Left*). This enhancement of sensitivity was also observed in the Clone K-TCR-transduced CD4-2D3 cells (Fig. 1c, *Right*). These results suggested that CD4-2D3 cells should be useful as platform cells for the evaluation of functional avidity of HLA class II-restricted TCRs.

### CD4-2D3 is a platform cell line for the evaluation of functional avidity of HLA class II-restricted TCRs

**Fig. 2:**
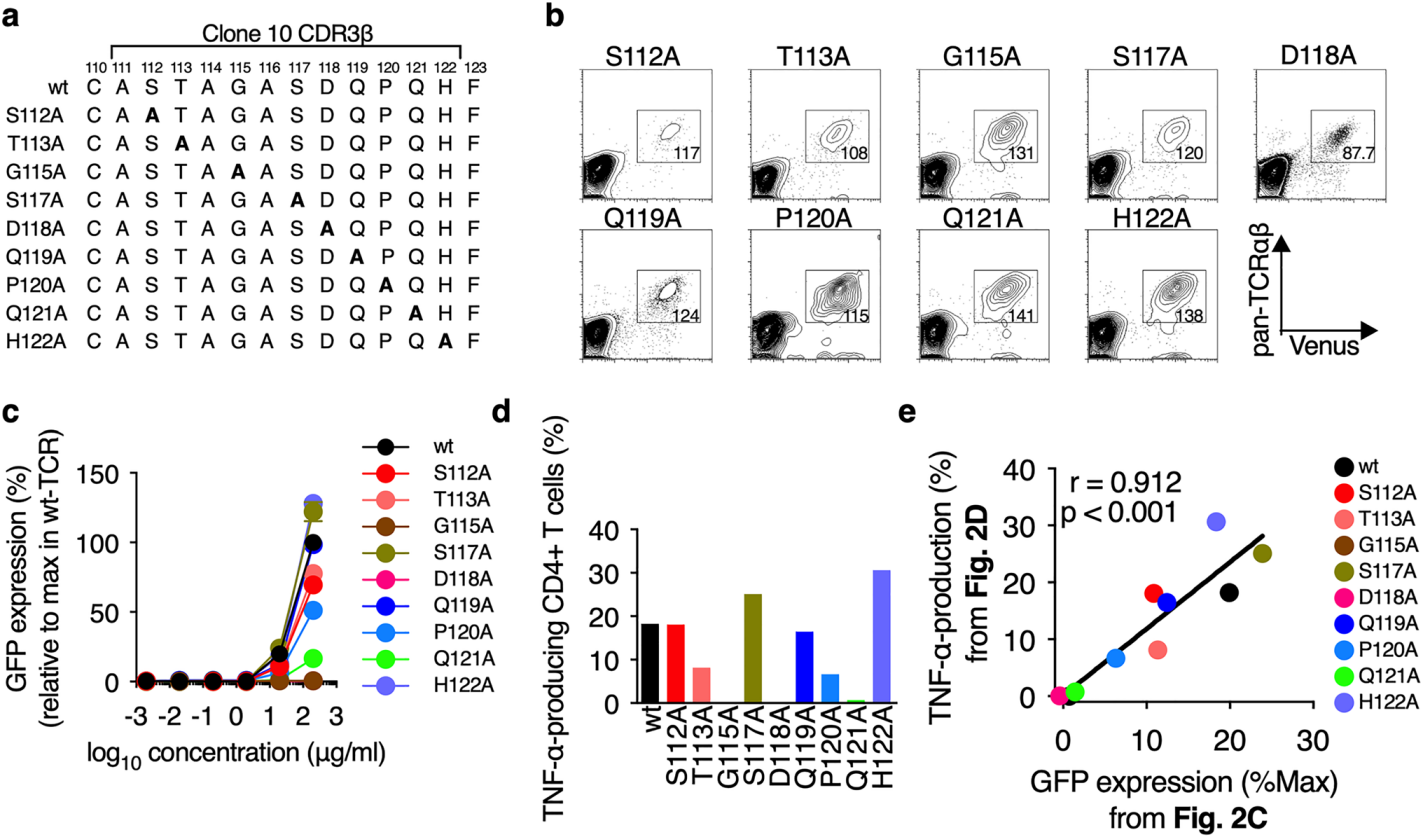
Functional correlation between TCR-transduced CD4-2D3 and CD4^+^ T cells. **a** CDR3β amino acid sequences of wild-type TCR (wt) and muTCRs. Bold A in the sequences shows a substituted alanine. **b** Dot plots showing TCR and Venus in the muTCR-transduced CD4-2D3 cells. The numbers show TCR expression levels, MFIs, in each gated cell population. **c** Representative peptide concentration–response curves of wt-TCR- and muTCR-transduced CD4-2D3 cells. Each symbol shows mean value ± SD from duplicate wells. All data are normalized as a percent of the maximal frequency of GFP-positive cells in wt-TCR-transduced CD4-2D3 cells. **d** Frequencies of TNF-*α*-producing cells in TCR-transduced CD4^+^ T cells. TNF-α-producing cells were detected by intracellular cytokine staining after the stimulation with 20 μg/ml of WT1_332_. **e** Correlation between GFP expression in TCR-transduced CD4-2D3 cells (from **c**) and TNF-*α* production in TCR-transduced CD4^+^ T cells (from **d**). Out of many data in panel **c**, data obtained when stimulated with 20 μg/ml of WT1_332_ were used for this correlation analysis. Pearson correlation coefficient was used to analyze the correlation between both data

In order to confirm the usefulness of CD4-2D3 cell line as a platform cell line for the evaluation of functional avidity of HLA class II-restricted TCRs, the functional correlation between the CD4-2D3 and freshly isolated human CD4^+^ T cells, both of which were transduced with the same wild-type and mutated TCRs, was examined. We generated nine Clone 10-TCR mutants such as S112A-, T113A-, G115A-, S117A-, D118A-, Q119A-, P120A-, Q121A-, and H122A-TCR by alanine-scanning mutagenesis targeting CDR3β (i.e., CDR3 of *β* chain) of wild-type Clone 10-TCR (wt-TCR) (Fig. 2a). These mutant TCRs (muTCRs) were correctly expressed on CD4-2D3 cells by lentiviral transduction although there were some differences (mean = 120.1; SD = 16.2) in their expression levels (MFIs of pan-TCRαβ) (Fig. 2b). Next, we stimulated these cells with the HLA-DPB1*05:01-expressing stimulator cells in the presence of the various concentrations of WT1_332_ peptide and then investigated their responsiveness to the peptide. Both G115A- and D118A-TCR-transduced CD4-2D3 cells did not express GFP even at high concentration (200 μg/ml) of WT1_332_ peptide (Fig. 2c), indicating that G and D amino acid residues at positions 115 and 118, respectively, in Clone 10-TCR CDR3β should be critical for the responsiveness to WT1_332_ peptide. Five muTCRs (S112A-, T113A-, Q119A-, P120A-, and Q121A-TCR) -transduced CD4-2D3 cells showed lower responsiveness to WT1_332_ peptide than wt-TCR-transduced and 2 muTCRs (S117A- and H122A-TCR) -transduced CD4-2D3 cells. Importantly, the difference in the responsiveness did not correlate (*r* = 0.24, *p* = 0.56, data not shown) with the TCR-expression levels, as described in Fig. 2b, and therefore it likely resulted from the difference in the TCR affinity to WT1_332_ peptide/HLA-DPB1*05:01 complex. To investigate functional correlation between muTCRs-transduced CD4-2D3 cells and freshly isolated human CD4^+^ T cells, we generated muTCR- and wt-TCR-transduced CD4^+^ T cells by lentiviral transduction and measured the frequencies of TNF-α-producing cells after stimulation of these cells with WT1_332_ peptide (Fig. 2d). As expected, there was a clear correlation (*r* = 0.912, *p* < 0.001) between the frequencies of GFP-expressing cells in the muTCR-transduced CD4-2D3 cells and TNF-α-producing cells in the muTCR-transduced CD4^+^ T cells (Fig. 2e). Taken together, these results demonstrate that CD4-2D3 cell line is useful as a platform cell line for the evaluation of the functional avidity of HLA class II-restricted TCRs.

### Functional avidity of wild and mutant Clone 10 TCRs

**Fig. 3:**
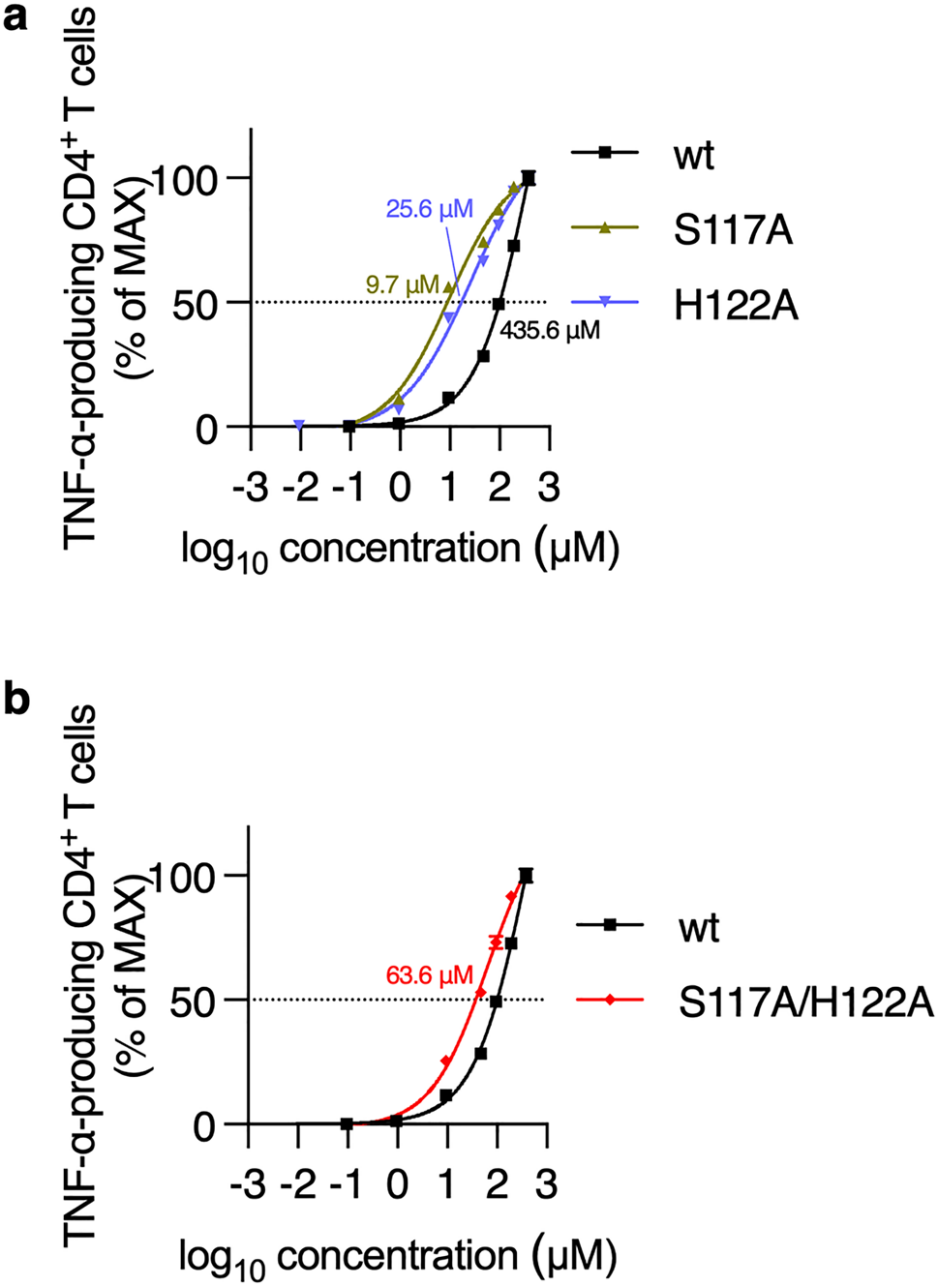
S117A-TCR showed the highest functional avidity in CD4^+^ T cells. **a** Representative peptide concentration–response curves of wt-TCR, S117A-TCR, and H122A-TCR-transduced CD4^+^ T cells. **b** Representative peptide concentration–response curves of wt-TCR and S117A/H122A-TCR-transduced CD4^+^ T cells. These experiments were performed two times and similar results were obtained

Next, functional avidities of wt-, S117A-, and H122A-TCR in CD4^+^ T cells were determined by calculating half maximal effective concentration (EC50) from WT1_332_ peptide concentration–response curves. EC50s were 9.7 μM (95% confidence interval (CI), 6.6–16.1 μM) for S117A-TCR, 25.6 μM (95% CI, 16.4–51.1 μM) for H122A-TCR, and 435.6 μM (95% CI, 221.0–1965 μM) for wt-TCR, revealing that alanine substitution at positions 117 and 122 in Clone 10-TCRβ increased the functional avidity of Clone 10-TCR to WT1_332_ peptide (Fig. 3a). These findings gave rise to the possibility that simultaneous substitution of S117A and H122A further increased the functional avidity. However, EC50 of the S117A/H122A TCR was 63.6 μM (95% CI, 45.6–107.7 μM), and its functional avidity was lower than that of one each of S117A- and H122A-TCR (Fig. 3b). Furthermore, we tried to isolate muTCRs with higher functional avidity than that of S117A-TCR, instead of alanine-scanning mutagenesis, by Clone 10-TCR mutation library, in which a degenerate codon, NNS (N = A/T/C/G, S = C/G) codon that encoded all 20 amino acids and decreased a probability of insertion of stop codons, was used for the substitution of amino acid residues at positions 117 and 122 in Clone 10-TCR*β*. However, we could not isolate such Clone 10-mutants although muTCRs with higher functional avidity than that of wt-TCR were successfully isolated (Supplementary Fig. 1). Therefore, S117A-TCR had the highest functional avidity among Clone 10-wild and -mutant TCRs.

### Potent cytotoxicity of S117A-TCR-transduced CD4^+^ T cells against WT1-expressing leukemia cells

**Fig. 4:**
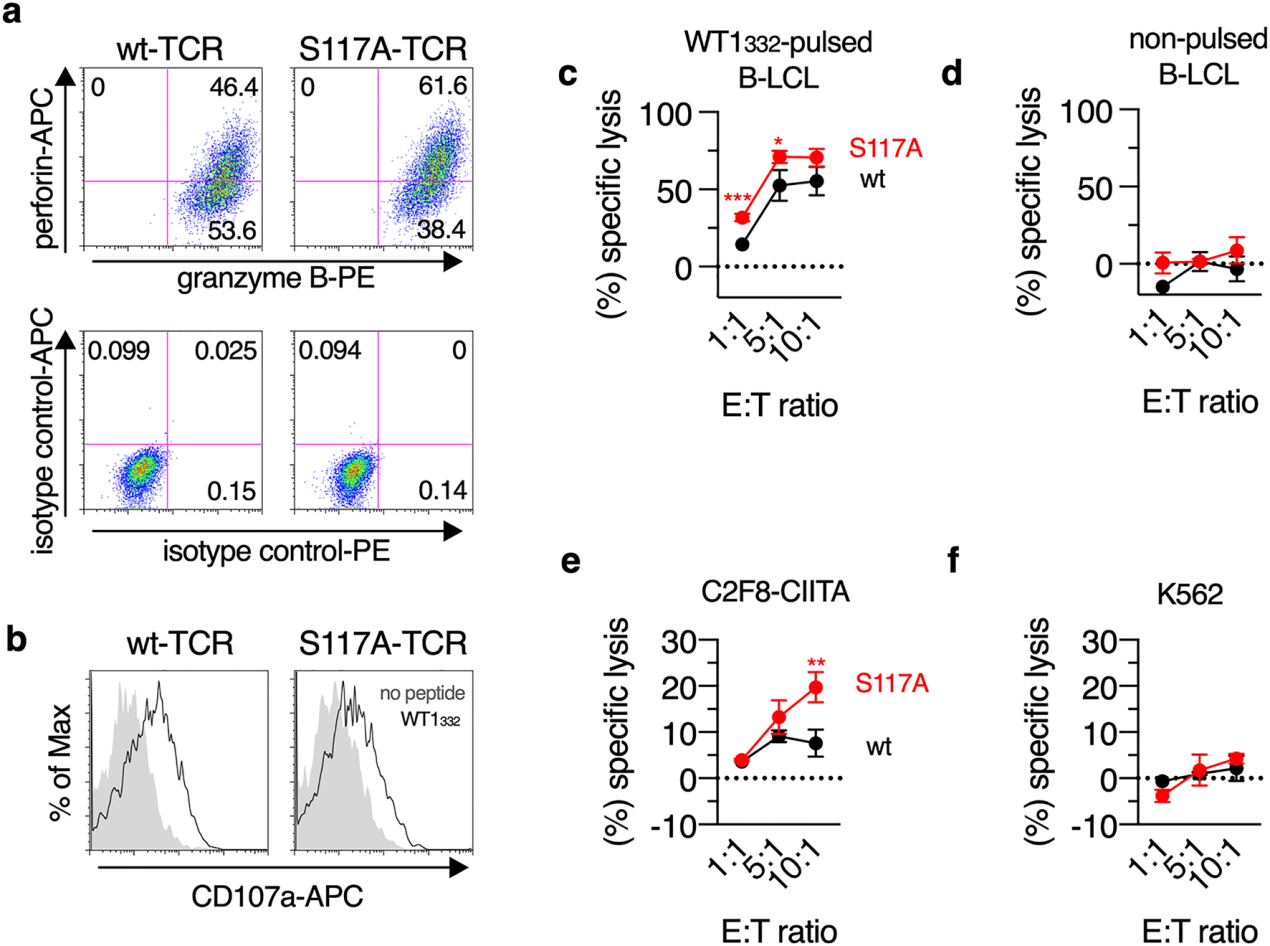
Transduction of S117A-TCR into CD4^+^ T cells conferred potent cytotoxic activity. **a** Representative dot plots showing granzyme B and perforin expression in TCR-transduced CD4^+^ T cells 3 days after in vitro stimulation with WT1_332_. **b** CD107a degranulation was detected in TCR-transduced CD4^+^ T cells after the stimulation with or without WT1_332_. Representative histograms are shown. **c**-**f** Killing activity against WT1_332_-pulsed B-LCL (**c**), non-pulsed B-LCL (**d**), C2F8-CIITA (**e**), or K562 (**f**) in TCR-transduced CD4^+^ T cells. Asterisks show a significant difference in (%) specific lysis between wt-TCR and S117A-TCR-transduced CD4^+^ T cells. *p < 0.05; **p < 0.01; ***p < 0.001 (unpaired *t*-test). Each symbol shows mean value ± SD from triplicate wells. Representative data are shown

In previous our study, wt-TCR-transduced CD4^+^ T cells showed cytotoxicity against WT1-expressing leukemia cells in an HLA-DPB1*05:01-restricted manner [4]. We next investigated whether cytotoxicity of TCR-transduced CD4^+^ T cells correlated with their TCR functional avidity. Human CD4^+^ T cells acquire cytotoxicity through the expression of perforin/granzyme B that are induced by TCR-stimulation [22, 23]. It was therefore thought that CD4^+^ T cells with a high-avidity TCR expressed a higher amount of perforin and/or granzyme B through in vitro stimulation with their antigenic peptides than CD4^+^ T cells with a low-avidity TCR. As shown in Fig. 4a, perforin and granzyme B were detected in both wt-TCR- and S117A-TCR-transduced CD4^+^ T cells 3 days after the second in vitro stimulation with WT1_332_, but the expression levels of perforin were higher in S117A-TCR-transduced CD4^+^ T cells than wt-TCR-transduced ones. In addition, both TCR-transduced CD4^+^ T cells expressed a degranulation marker, CD107a on their surface in response to WT1_332_ peptide (Fig. 4b), indicating that these CD4^+^ T cells could exert cytotoxicity. In fact, both TCR-transduced CD4^+^ T cells effectively lysed WT1_332_ peptide-pulsed B-LCL and WT1-expressing HLA-DPB1*05:01-positive leukemia cell line, C2F8-CIITA (Fig. 4c and e). On the other hand, they lysed neither WT1_332_ peptide-non-pulsed B-LCL nor HLA-DPB1*05:01-negative WT1-expressing leukemia cell line, K562 (Fig. 4d and f). Importantly, S117A-TCR-transduced CD4^+^ T cells showed more potent cytotoxicity compared to wt-TCR-transduced CD4^+^ T cells (Fig. 4c and e). Furthermore, the higher cytotoxic capacity in S117A-TCR-transduced CD4^+^ T cells was observed even when another healthy donor was used as a source of TCR-transduced CD4^+^ T cells (Supplementary Fig. 2). These results showed the possibility that the TCR functional avidity should correlate with cytotoxicity of the CD4^+^ T cells transduced with the same TCR. Thus, the highest-avidity TCR, S117A-TCR should be the most promising TCR for developing CD4^+^ T cell-based adoptive T cell therapy against HLA class II-positive WT1-expressing malignancies, such as leukemia.

## Discussion

In this study, we established a novel platform cell line, CD4-2D3 for the evaluation of functional avidity of HLA class II-restricted TCRs. By using this CD4-2D3 cell line, we will be able to standardize the method to evaluate TCR functional avidity of HLA class II-restricted TCRs and select the best TCRs for developing adoptive TCR-T cell therapy against cancer and infectious disease.

In general, HLA-matched primary T cells are often used for validating TCRs. However, the change of cytokine production [4, 21] and the down-regulation of TCR expression (unpublished data) in TCR-transduced CD4^+^ T cells often occurred during culture. Furthermore, the periodical stimulation indispensable for the maintenance of TCR-transduced CD4^+^ T cells induces cytokine production and the expression of activation markers such as CD25, CD69, and CD137, and as the result, the background in the TCR/antigen signaling assay rises and thus the results become incorrect. Therefore, primary T cells are not suitable for evaluating TCR function. On the other hand, CD4-2D3 cell line is useful to identify the antigens specific for the TCRs isolated from the CD4^+^ T cells that dominantly and clonally existed in patients with cancer, infectious, inflammatory, or auto-immune diseases. Recent advances in high-throughput immune sequencing using next-generation sequencing technologies provides us with a huge number of TCR α- and β-chain repertoires but not with the information of which TCR α- and β-chains combine to form functional TCR specific for an antigen. To overcome this problem, Howie et al. developed a new method called pairSEQ [24]. PairSEQ provided in silico information of TCR pairs from the thousands to hundreds of thousands of T cells taken from peripheral blood and tumors. Therefore, these TCR pairs needed to be validated (had to be validated) in vitro whether they were functional TCRs and what antigen they recognized [25]. For the validation of these TCR pairs, CD4-2D3 cell line is very useful. In conclusion, first, CD4-2D3 cell line will make it possible to identify the antigens specific for the antigen-unknown TCRs isolated from cancer, inflammatory, and autoimmune lesions. Second, CD4-2D3 cell line should provide for the methodology to mature and optimize TCR avidity for clinical application. TCR avidity maturation had often been performed in order to develop high-avidity HLA class I-restricted TCRs for clinical use [26–28]. These studies mainly used HLA class I-tetramer to screen the TCRs with high avidity to the corresponding antigen. On the other hand, to screen high-avidity HLA class II-restricted TCRs, HLA class II-tetramers (or -multimers) are not available in many cases because of a lack of evidence showing the correlation between TCR-binding strength to multimers and CD4^+^ T cell function [29]. Therefore, instead of HLA class II-multimers, platform cell lines that can directly link to CD4^+^ T cell function are being demanded. In the present study, we successfully maturated TCR avidity of Clone10-TCR using targeted amino acid substitution (i.e., alanine-scanning mutagenesis) and isolated S117A-TCR with higher avidity than that of wt-TCR. In addition, taking advantage of GFP reporter that made it possible to detect antigen-reactive living cells at single-cell level, we applied CD4-2D3 cell line as a platform cell for library-based TCR screening and successfully isolated TCRs showing higher avidity to WT1_332_ than the avidity of wt-TCR (Supplementary Fig. 1). These results demonstrate that CD4-2D3 cell line is a platform cell line not only to evaluate functional avidity of HLA class II-restricted TCRs but also to high-throughput screen HLA class II-restricted TCRs with higher avidity.

In CD8^+^ T cells, it is well-known that cytotoxicity is generally correlated with TCR avidity [9]. However, it remains unclear whether or not the same is true in CD4^+^ T cells. Since human CD4^+^ T cells require TCR-stimulation to express granzyme and perforin [22, 23], the TCR avidity is likely to correlate with the cytotoxicity in CD4^+^ T cells. In fact, a high-avidity TCR, S117A-TCR-transduced CD4^+^ T cells had a higher frequency of granzyme B/perforin-double positive cells and showed more potent cytotoxicity against WT1-expressing and HLA-DPB1*05:01-positive leukemia cells, compared to wt-TCR-transduced CD4^+^ T cells. However, Cachot et al. analyzed numerous CD4^+^ T cell clones isolated from melanoma patients and showed that some CD4^+^ Th and CTL clones shared identical TCRs with the same avidity [7]. These findings indicated that the cytotoxicity of CD4^+^ T cells was not determined only by the TCR avidity. The CD4^+^ T cell cytotoxicity seems to be determined by both the TCR avidity and micro-environmental cytokines. Similarly, the polarizing to Th1 and Th2 cells is also determined by both the TCR avidity and micro-environment as drivers [30, 31]. Stronger and weaker TCR signals induce Th1 and Th2 cell differentiation, respectively, but this differentiation mechanism is not definitive and is affected by the micro-environmental cytokines, such as IL-12, IFN-γ, and IL-4. Therefore, the determination of the correct TCR avidity by using CD4-2D3 cell line should make it possible to analyze what kinds of drivers affected the differentiation of the avidity-determined TCR-expressing CD4^+^ T cells into CD4^+^ CTL.

Finally, in the present study, we have successfully established CD4-2D3 cell line useful for rapid, robust, and standardized evaluation of HLA class II-restricted TCRs. This platform cell line is expected to accelerate the development of TCR-T cell therapy against cancers. However, this platform cell line does not reflect and anticipate all function of TCR-transduced CD4^+^ T cells. So far, we have experienced that some isolated TCRs do not effectively express on primary CD4^+^ T cells to induce their function fully, and that other TCRs work well more than we expected. Therefore, we should not skip functional analysis of TCR-transduced CD4^+^ T cells during the process from selecting optimal TCRs by using CD4-2D3 to clinical trials.

## Supplementary Information

Below is the link to the electronic supplementary material.

Supplementary Figure1 (TIFF 33977 kb)

Supplementary Figure2 (TIFF 33977 kb)

## Abbreviations

CIITA: Class II major histocompatibility complex transactivator
GFP: Green fluorescent protein
HLA: Human leukocyte antigen
IFN: Interferon
IL: Interleukin
MHC: Major histocompatibility complex
NFAT: Nuclear factor of activated T cells
TCR: T cell receptor
Th1: T helper cell type 1
Th2: T helper cell type 2
TNF: Tumor necrosis factor
Treg: Regulatory T cell
