# Supplementary figures and images for: Establishment of a novel NFAT-GFP reporter platform useful for the functional avidity maturation of HLA class II-restricted TCRs
Source: Cancer Immunol Immunother. 2023 Mar 20;72(7):2347–56. doi: 10.1007/s00262-023-03420-8 (PMC10264488; doi:10.1007/s00262-023-03420-8)

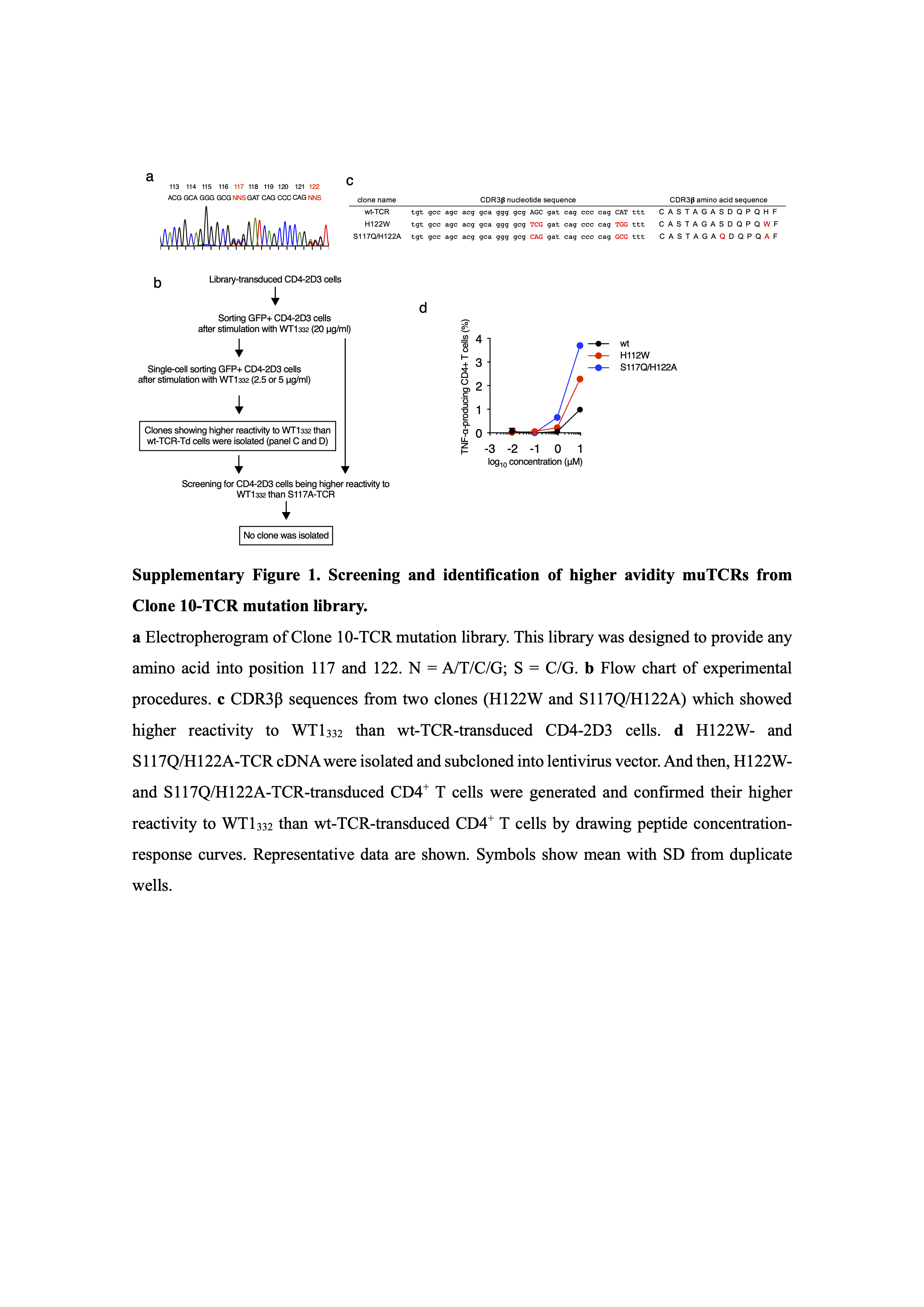

Supplement: Supplementary file 1 — Supplementary Figure1 (TIFF 33977 kb) [file 262_2023_3420_MOESM1_ESM.tiff]

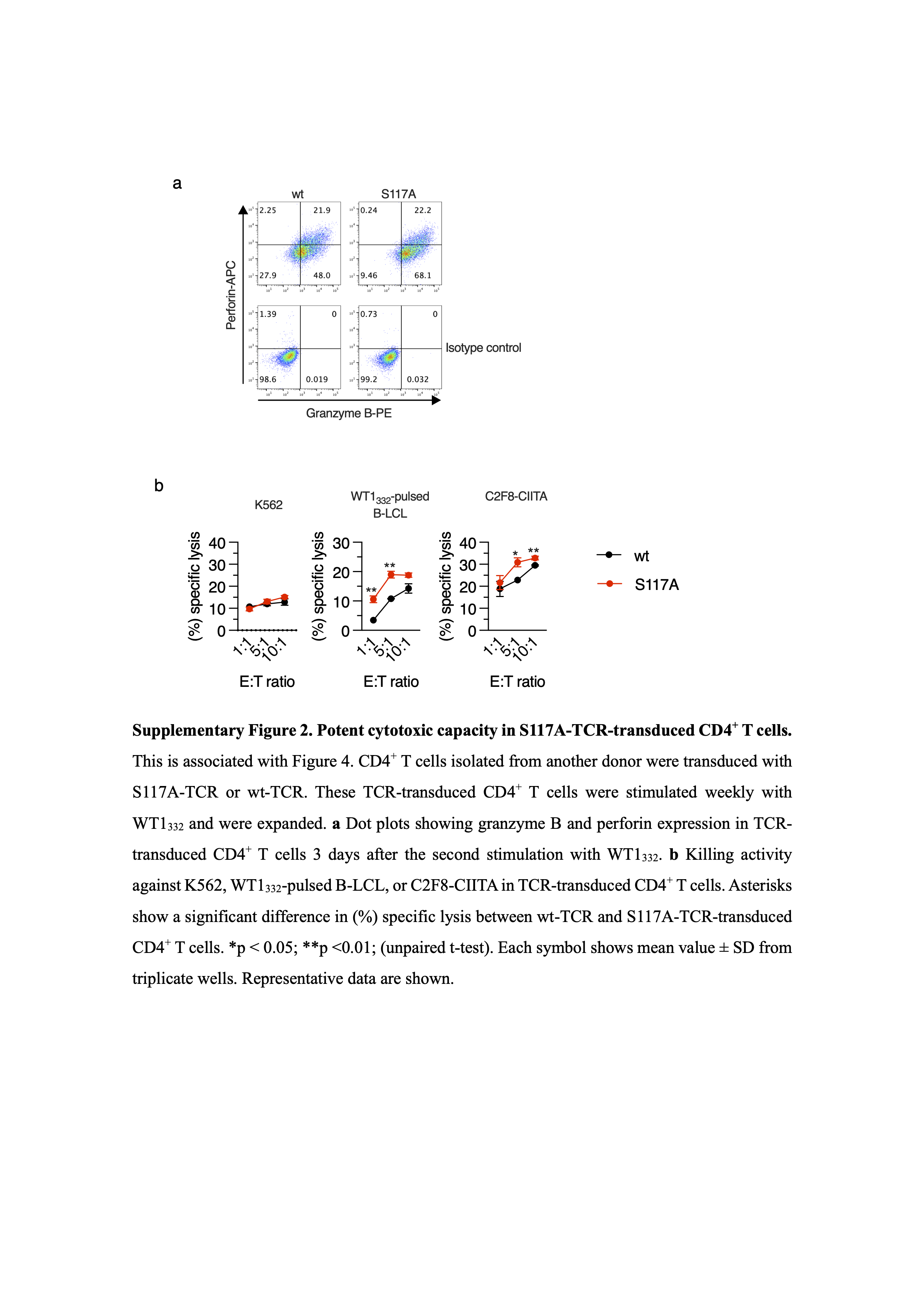

Supplement: Supplementary file 2 — Supplementary Figure2 (TIFF 33977 kb) [file 262_2023_3420_MOESM2_ESM.tiff]
